# Piece by piece: making plant natural products accessible via heterologous biosynthesis

**DOI:** 10.1093/synbio/ysac028

**Published:** 2022-11-08

**Authors:** Kira J Tiedge

**Affiliations:** Groningen Institute for Evolutionary Life Sciences, University of Groningen, Groningen, The Netherlands

Numerous of the products we use in our daily lives originally come from plants. These plant-derived natural products include flavors, fragrances and medicines, such as the fragrance limonene from citrus fruits or the antimalarial drug artemisinin. As we cannot grow enough plants to satisfy our demand for these compounds, researchers are trying to manufacture these natural products in their laboratories via expression in easy-to-culture plants, bacteria or yeast—so-called heterologous hosts. Like this, researchers can create cell factories that can make more than what is made by the natural host. For smaller molecules like limonene, this is a fairly streamlined process as only one enzyme needs to be added to a host to create such a cell factory (1). However, for making chemically more complex bioactive molecules, adding 10 or more enzymatic reactions is required. Cloning these reactions being hard enough, the real bottleneck for making chemically complex natural products is the fact that the enzymes catalyzing the biosynthesis are often not known and need to be identified first. Recently, a group of researchers from the Max Planck Institute for Chemical Ecology in Jena, Germany, managed to decipher the multistep biosynthetic pathway of strychnine, a toxic alkaloid which is famously used as poison in crime stories and as a pesticide in real-world applications. Furthermore, they were able to transfer all required precursor enzymes as well as nine newly identified enzymes together into a heterologous host for transient expression (2), delivering a blueprint for creating cell factories able to perform complex plant chemistry.

Hong *et al.’s* achievement is remarkable for several reasons: plants have very complex genomes, making it hard to identify which genes are encoding the biosynthesis of a desired product. For example, a plant genome can host many gene candidates that could act as the code for a specific enzymatic reaction. Finding out which is the right one involves a laborious screening process: the selected genes need to be cloned into an expression vector and then transformed into an expression system, such as tobacco plants (*Nicotiana benthamiana*), where their catalytic activity can be confirmed. Although advances in deoxyribonucleic acid (DNA) synthesis have helped in overcoming bottlenecks in cloning plant DNA (3), many challenges of identifying all the puzzle pieces that allow a plant to make a desired product and putting them together in the right order remain.

The complex biosynthetic pathway of strychnine had puzzled the field for more than two centuries since its first isolation from the strychnine tree (*Strychnos nux-vomica*; 4). The route to success in the work by Hong *et al.* was a combination of integrating large -omics datasets, chemical logic and high-throughput functional enzyme characterization. To find the potentially involved enzymes, the researchers combined knowledge about metabolite abundance in different tissues with transcriptomic data from the same samples to identify genes that are highly co-expressed under high-abundance conditions. To prove that they had found the correct enzymes, the researchers step-by-step co-expressed an increasing combination of enzymes together in tobacco plants, eventually deciphering the pathway piece by piece. Ultimately, they were able to get the plants to produce strychnine and other related strychnos alkaloids with potential industrial relevance.

Identification of a biosynthetic pathway is the first step in building cell factories that can make a lot of the desired compound and the next step is optimizing the production. Here, recent advances in metabolic engineering have showcased the creation of optimized cell factories for the production of other important plant molecules such as monoterpene indole alkaloids, used to treat cancer and malaria (5), or the chemotherapy compound vinblastine (6).

Taken together, these advances in identifying complex biosynthesis pathways in plants and optimizing their performance in heterologous hosts have the potential to open new avenues for cracking the vast number of unsolved puzzles in natural product biosynthesis, especially when combined with capacities in artificial intelligence for protein prediction and engineering (7), open-source and open-data management practices (8) and the design of cell-free expression systems (9). However, the future needs to show if production can be scaled up to an industrially relevant level, but we are certainly on the right track to better explore and utilize the rich diversity of plant natural products for human health, food and agricultural applications.
